# Personal

**Published:** 1900-02

**Authors:** 


### PERSONAL.

Dr. C. B. Burr, of Flint, Mich., medical superintendent of the Oak Grove Hospital for the Insane completed his twenty-first year of hospital work November 24, 1899, on which occasion a delightful surprise was arranged by his friends, who presented him with a loving cup. The presentation speech was made by Dr. E. A. Christian, superintendent of the Eastern Michigan Asylum, at Pontiac, in the presence of Dr. Burr's friends from various parts of the state who were present to do him honor. Five years ago he went to Flint to take charge of Oak Grove Sanitarium, previous to which time he had been for several years in charge of the Eastern Michigan Hospital, at Pontiac. Dr. Burr has a national reputation as an alienist.

---

Dr. Edward J. Ill, of Newark, N. J., will read a paper on Indications for and the selection of operation for uterine myomata, before the gynecological section of the Buffalo Academy of Medicine, Tuesday evening, February 27, 1900. Dr. Ill is one of the most accomplished gynecological operators of the period, and a full attendance is anticipated on the occasion of his visit.

Dr. Charles T. St. John, of Buffalo, as resigned as house-surgeon of Riverside Hospital to accept an appointment as Acting Assistant Surgeon U. S. Army. He has been ordered to proceed to the Philippines immediately by way of San Francisco.

---

Dr. A. H. Doty, health officer of the port of New York, paid Buffalo a short visit January 18, 1900. He called on Health Commissioner Wende and accompanied him to the garbage crematory in Cheektowaga. Dr. Doty is making himself familiar with Buffalo's system of disposing of its garbage. He took an afternoon train east after the inspection.

---

Dr. Thomas M. Heard, Jr., of 147 Lafayette Avenue, Buffalo, has been appointed house surgeon for the Riverside Hospital, vice Dr. St. John resigned. This appointment is a commendable one and we predict benefit to all interested as a result.

---

Dr. O. C. Shaw, for many years a resident of Kennedy, Chautauqua County, has removed to Hamburg, N. Y. Dr. Shaw receives a warm welcome in Erie County where he is well known as a competent practitioner of medicine.

---

Dr. B. C. Loveland, formerly of Clifton Springs, N. Y., has removed to Syracuse. He has established himself in the active practice of his profession at 608 South Salina Street, in that city.

